# 

**Published:** 1888-05

**Authors:** 


### TABLE OF CONTENTS.

### Original and Selected Articles.

What is the matter with me, and what shall I do? by George C. Spearman, M.D., of Pennington, Ga........ 161
Preventive treatment of suppurative inflammation of the middle ear, by R. O. Cotter, M.D., Macon, Ga........ 164
Inflammation of the neck of the bladder, by Jno. Thad. Johnson, M. D........ 166
Chronic syphilis, by H. M. Lawson, M. D, Cuthbe[illegible]t, Ga; hypnotism in therapeutics... 171
Hypodermic injections of antipyrine in painful diseases, by S. Fraenkerl, M. D., in "Deutsche Med. Wochenschrift"........ 173
Cerebro spinal meningitis and its treatment... 175
Diphtheria........ 176

### Abstracts and Gleanings.

Asclepias syriaca (milkweed) for lumbago and dropsy........ 177
Two cases of placenta prævia........ 178
Concerning chloroform; whooping cough..... 179
Fistula in ano........ 180
New remedy for cystitis........ 181
The Mutual relation between the saliva and the gastric juice; abating typhoid fever....... 182
Hydrocele; Hydrate of Amylene........ 183
Apomorphia; how to grow fat; salicylate of soda........ 184
Acid treatment of intestinal diseases of infancy and childhood; tying the umbilical cord........ 185
How to treat cramps in the leg; antipyrin as homestatic and disinfectant; a successful treatment of hydrophobia........ 186
Ague; embalming fluid; treatment of ulcers of the leg........ 187
Red hawthorn in uterine hemorrhage; rapid and simple method of reducing downward dislocation of the shoulder; erythrophleine; remedy for epithelioma........ 188
Permanganate of potassium in toothache; menthol and mentholeata; a new local anæsthetic; the treatment of post-partum hemorrhage; treatment of ingrowing toe-nail... 189
An important incompatibility; antipyrin in nocturnal emissions; sozoidol; hip dislocations; salol in rheumatism........ 190

### Scientific Items.

Who is never crazy; simple methods for reviving unconscious persons........ 191
Light from the stars; the sinking Cordillera of the Andes; remote stars........ 192

### Practical Notes and Formulæ.

Salicylic collodium; insect stings; formulæ for purgative mixtures........ 193
Agaricin phthisis; for inhalation in phthisis; a laxative gastronic; a mouth wash; pain obtunder after extraction........ 194

### Editorials and Miscellaneous.

Editoral brevities........ 195
The case of Dr. Spearman........ 196
Substitution........ 197
Medical Association of Georgia........ 198
Special notes........ 200

